# The pUL37 tegument protein guides alpha-herpesvirus retrograde axonal transport to promote neuroinvasion

**DOI:** 10.1371/journal.ppat.1006741

**Published:** 2017-12-07

**Authors:** Alexsia L. Richards, Patricia J. Sollars, Jared D. Pitts, Austin M. Stults, Ekaterina E. Heldwein, Gary E. Pickard, Gregory A. Smith

**Affiliations:** 1 Department of Microbiology-Immunology, Northwestern University Feinberg School of Medicine, Chicago, Illinois, United States of America; 2 School of Veterinary Medicine and Biomedical Sciences, University of Nebraska-Lincoln, Lincoln, Nebraska, United States of America; 3 Department of Molecular Biology and Microbiology, Tufts University School of Medicine, Boston, Massachusetts, United States of America; 4 Department of Ophthalmology and Visual Sciences, University of Nebraska Medical Center, Omaha, Nebraska; Geisel School of Medicine at Dartmouth, UNITED STATES

## Abstract

A hallmark property of the neurotropic alpha-herpesvirinae is the dissemination of infection to sensory and autonomic ganglia of the peripheral nervous system following an initial exposure at mucosal surfaces. The peripheral ganglia serve as the latent virus reservoir and the source of recurrent infections such as cold sores (herpes simplex virus type I) and shingles (varicella zoster virus). However, the means by which these viruses routinely invade the nervous system is not fully understood. We report that an internal virion component, the pUL37 tegument protein, has a surface region that is an essential neuroinvasion effector. Mutation of this region rendered herpes simplex virus type 1 (HSV-1) and pseudorabies virus (PRV) incapable of spreading by retrograde axonal transport to peripheral ganglia both in culture and animals. By monitoring the axonal transport of individual viral particles by time-lapse fluorescence microscopy, the mutant viruses were determined to lack the characteristic sustained intracellular capsid motion along microtubules that normally traffics capsids to the neural soma. Consistent with the axonal transport deficit, the mutant viruses did not reach sites of latency in peripheral ganglia, and were avirulent. Despite this, viral propagation in peripheral tissues and in cultured epithelial cell lines remained robust. Selective elimination of retrograde delivery to the nervous system has long been sought after as a means to develop vaccines against these ubiquitous, and sometimes devastating viruses. In support of this potential, we find that HSV-1 and PRV mutated in the effector region of pUL37 evoked effective vaccination against subsequent nervous system challenges and encephalitic disease. These findings demonstrate that retrograde axonal transport of the herpesviruses occurs by a virus-directed mechanism that operates by coordinating opposing microtubule motors to favor sustained retrograde delivery of the virus to the peripheral ganglia. The ability to selectively eliminate the retrograde axonal transport mechanism from these viruses will be useful in trans-synaptic mapping studies of the mammalian nervous system, and affords a new vaccination paradigm for human and veterinary neurotropic herpesviruses.

## Author summary

Neuroinvasive members of the alpha-herpesvirinae include human (i.e. herpes simplex virus type I; HSV-1) and veterinary (i.e. pseudorabies virus; PRV) pathogens that routinely invade the peripheral nervous system of an immunocompetent host in the absence of overt tissue damage. We have identified an essential, and conserved, component of these viruses that directs incoming viral particles into the neural ganglia. Viruses carrying mutations in this protein effector region propagate normally at peripheral sites of inoculation but fail to invade the nervous system by retrograde axonal transport, cannot establish life-long latent infections, and are avirulent. These properties define a promising new class of live-attenuated vaccines that protect from subsequent nervous system invasion and encephalitic disease.

## Introduction

Neuroinvasive members of the alpha-herpesvirinae include human (i.e. herpes simplex virus type I; HSV-1] and veterinary (i.e. pseudorabies virus; PRV) pathogens that establish life-long latent infections in neurons of the peripheral nervous system (PNS) [1]. Infections by these viruses can be subtle or manifest in severe disease, the latter ranging from herpes simplex encephalitis in humans to Aujeszky’s disease in swine [2,3]. Unlike other neurotropic viruses, the neuroinvasive alpha-herpesvirinae routinely access the nervous system, and do so in the absence of obvious tissue trauma. The robust nature of the invasive process is evidenced by the presence of herpesvirus DNA in the nuclei of peripheral neurons in most adults [4]. Deciphering the mechanisms underlying virus delivery from the mucosa to neuronal cell bodies of sensory and autonomic ganglia is of interest not only for its remarkable biology, but also to exploit it as a gene delivery vehicle and to produce a new generation of live-attenuated vaccines [5,6].

The alpha-herpesvirinae are unusual among neurotropic viruses in that upon entering nerve endings they do not rely on endosomes for axonal trafficking to neuronal cell bodies [7]. Instead, these viruses fuse their envelopes with a cellular membrane to deposit the 125 nm diameter icosahedral capsid into the cytosol. An internal protein matrix surrounding the capsid in the virion, referred to as the tegument, is deposited in the cytosol with the capsid [8–12]. Although the majority of the tegument disassociates from the capsid at this stage, three tegument proteins remain capsid bound: pUL36, pUL37, and pUS3 [13–17]. Deciphering the contributions of pUL36 and pUL37 during initial infection is made complicated by their subsequent roles during virus assembly and egress; eliminating either tegument protein severely compromises production of HSV-1 and PRV virions [18–28].

The pUL36 tegument protein is directly anchored to the capsid surface [29] where it promotes trafficking to the nucleus by tethering the capsid to the dynein/dynactin microtubule motor complex [30], and by docking the capsid at a nuclear pore and triggering genome release [31–39]. pUL36 is also a neuroinvasive factor, which uses ubiquitination as a molecular switch to promote virus spread into the nervous system [40–42]. Less is known about pUL37 and its contribution to neuroinvasion. Like pUL36, pUL37 promotes translocation of incoming capsids to the nucleus [43]. The HSV-1 version of the protein is a deamidase and may increase cell permissivity to infection through interactions with TNF receptor associated factor 6 (TRAF6), melanoma differentiation-associated protein 5 (MDA5), and retinoic acid-inducible gene I (RIG-I); although residues associated with deamidation and TRAF6 binding are not conserved across the neuroinvasive herpesviridae [44,45]. Only a null mutant has been extensively examined *in vivo*, and although the mutant was impaired for invasion of the nervous system, this defect was more generally associated with a broad inability to propagate in all tissues examined [46]. We recently determined the crystal structure of the N-terminal half of pUL37 and identified three evolutionarily conserved surface-exposed regions: R1, R2 and R3 [47]. The three regions are distal to the portion of pUL37 that is associated with deamidase activity, TRAF6 binding, and interactions with other virion structural proteins [44,45,48,49]. Although these N-terminal regions were dispensable for replication in an epithelial cell line [47], in this report we demonstrate that one of these regions, R2, is critical for invasion of the nervous system.

Mutation of R2 in representative members of the two neuroinvasive genera of the alpha-herpesvirinae, PRV (varicellovirus genus) and HSV-1 (simplexvirus genus) in both instances eliminated viral invasion of the PNS *in vivo*. Time-lapse imaging of cultured primary sensory neurons following infection with fluorescent reporter viruses revealed that mutations in R2 profoundly impaired retrograde axonal transport. This defect was not due to a loss of capsid intracellular transport but rather stemmed from a loss of targeted transport. Capsids of the mutant viruses engaged in aberrant bidirectional motion that did not support long-distance axonal trafficking to neural soma. In contrast, R2 was dispensable for axonal transport during the egress phase of neuronal infection, with virions delivered to distal axons at the same efficiency as wild-type infections, and indicating that axonal transport at these two stages of infection occurs by independent mechanisms. These results define a critical component of the viral apparatus that mediates the neuroinvasion of the alpha-herpesviruses and indicate that the R2 region enables sustained retrograde transport to neural soma.

The ability to selectively eliminate the neuroinvasive property of these viruses, without otherwise impairing their replication, has potential as a new class of live-attenuated vaccines. In principle, the selective attenuation afforded by the R2 mutants should prevent vaccine-associated neuroinvasive disease and life-long latent infections, which would otherwise become problematic if the individual’s immune system were to later become compromised, while simultaneously preserving robust replication and spread in peripheral tissues required to elicit a pronounced immune response. To this end, we include an initial demonstration that R2-mutant viruses efficiently protect from wild-type HSV-1 and PRV challenges and offer robust protection from ocular disease, nervous system invasion, and encephalitis.

## Results

### R2 is an essential virulence determinant

**Fig 1:**
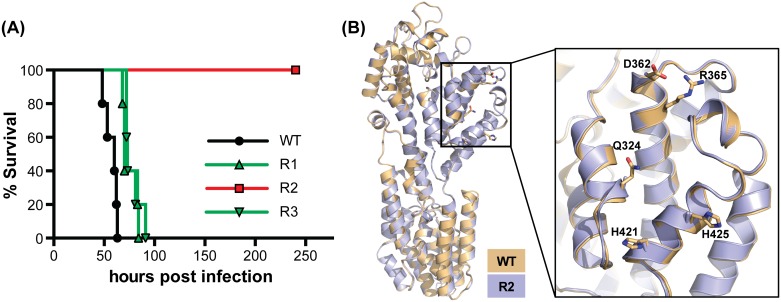
The PRV pUL37 R2 region is essential for virulence and can be mutated without causing misfolding of the surrounding protein structure. **(A)** Kaplan–Meier presentation of mouse survival following intranasal instillation of wild-type PRV (WT) or PRV carrying mutations in the R1, R2, or R3 regions of the pUL37 tegument protein (n = 5 animals for each virus). All viruses encode a mCherry tag fused to the pUL25 capsid protein as previously described [30,47]. **(B)** The crystal structure of the N-terminal half of the PRV pUL37 R2 mutant (R2; lilac), determined in this work, was overlaid onto the previously determined wild-type structure (WT; beige) [47] with rmsd 0.5538 Å over 479 aligned residues as determined in Coot [93]. A close-up view of R2 is shown to the right with the side chains of the five targeted amino acids indicated for wild type and the mutant.

We previously mutated three evolutionary conserved surface regions of the pUL37 tegument protein (R1, R2, R3) in PRV [47]. Each mutated region consisted of five alanine substitutions (S1 Table). Although plaques produced by the R2 mutant were smaller than wild type, all three mutants propagated to wild-type titers [47]. To extend this characterization, each mutant virus was examined *in vivo* following intranasal instillation into mice. Whereas wild-type PRV caused rapid lethality, the derivatives of PRV mutated for R1 and R3 were attenuated. Remarkably, the R2 mutant was avirulent despite propagating to wild-type titers in culture (Fig 1A) [47]. To determine if the stark R2 mutant phenotype resulted from misfolding of the mutant pUL37 protein, the crystal structure of the pUL37 N-terminus was solved and overlaid with the previous WT structure [47]. The five point mutations did not affect the overall fold of the N-terminal half of pUL37 as judged by the alignment of the wild-type and R2 mutant crystal structures (rmsd = 0.55 Å) (Fig 1B). These findings demonstrated that introduction of the mutations did not contort the pUL37 backbone, and encouraged us to further examine the R2 mutant in cell culture infection models to identify the specific source of its avirulence.

### R2 promotes establishment of infection

**Fig 2:**
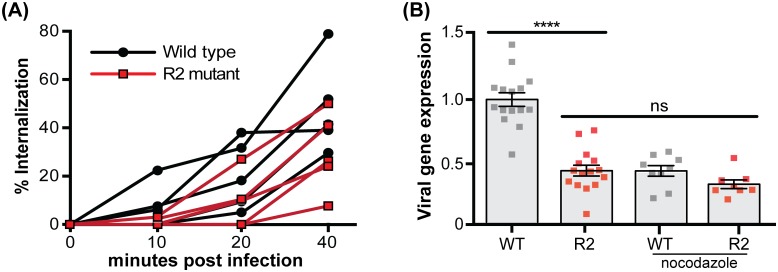
Mutation of PRV R2 results in a post-entry microtubule-dependent delay in gene expression. **(A)** Percent of PRV internalized into PK15 epithelial cells prior to extracellular virion inactivation with citrate at indicated times post-infection. Five experimental replicates are shown for each virus, with each representing average values from duplicate infections. **(B)** Expression of the PRV immediate early gene IE180 was quantified by qRT-PCR and normalized to expression of the host S28 rRNA at 4 hpi with the wild-type (WT) and R2 mutant (R2). Where indicated, 9 μM of nocodazole was added 1 hr prior to infection and maintained throughout the infection. All values were plotted relative to levels observed during the untreated wild-type (WT) infection. Three independent experiments were performed with each experimental replicate performed in triplicate. Values are expressed as mean ± s.e.m. (****, p < 0.0001; ns, not significant based on two-tailed unpaired *t* test).

Because the ability of the pUL37 R2 mutant to propagate to wild-type titers was inconsistent with an assembly or egress defect, we examined whether R2 contributed to the initiation of infection. Although PRV entry into epithelial cells was not significantly delayed by R2 mutation (Fig 2A), immediate-early viral gene expression was reduced (Fig 2B). The R2 mutant delay in viral expression was equivalent to the delay imposed on wild-type infections treated with nocodazole (a microtubule depolymerization drug), yet nocodazole treatment did not further reduce gene expression from the R2 mutant (Fig 2B). These results provided an initial indication that R2 functions at a post-entry microtubule-dependent step early in infection.

### R2 is a conserved effector of microtubule-based retrograde axonal transport

**Fig 3:**
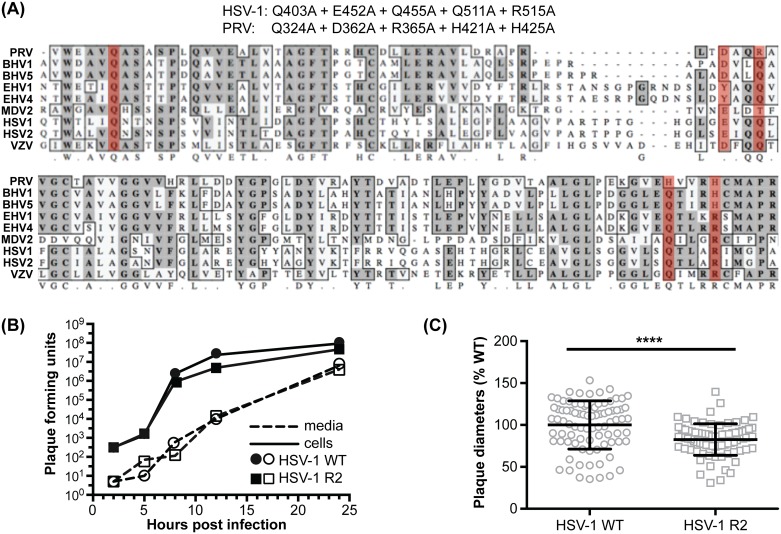
Mutation of the HSV-1 pUL37 R2 cluster phenocopies the PRV pUL37 R2 mutant. **(A)** Amino acid mutations resulting from five codon changes introduced into the UL37 gene of HSV-1 are indicated at top, along with the corresponding mutations of the previously described PRV R2 mutant and the respective positions of the mutations within a sequence alignment of nine alpha-herpesviruses (mutated positions highlighted in red). **(B)** Wild-type (WT) and R2-mutant (R2) HSV-1 single-step propagation kinetics were determined by counting plaque-forming units harvested from media and Vero epithelial cells (cells) at the times indicated. Viruses used in this study were not modified to encode a fluorescent tag. **(C)** Fluorescent plaque diameters of WT and R2-mutant HSV-1 encoding mCherry fused to the pUL25 capsid protein were compiled from three independent experiments and plotted as a percentage of the average WT plaque diameter. Error bars are s.d. (****, p < 0.0001 based on a two-tailed unpaired *t* test).

**Fig 4:**
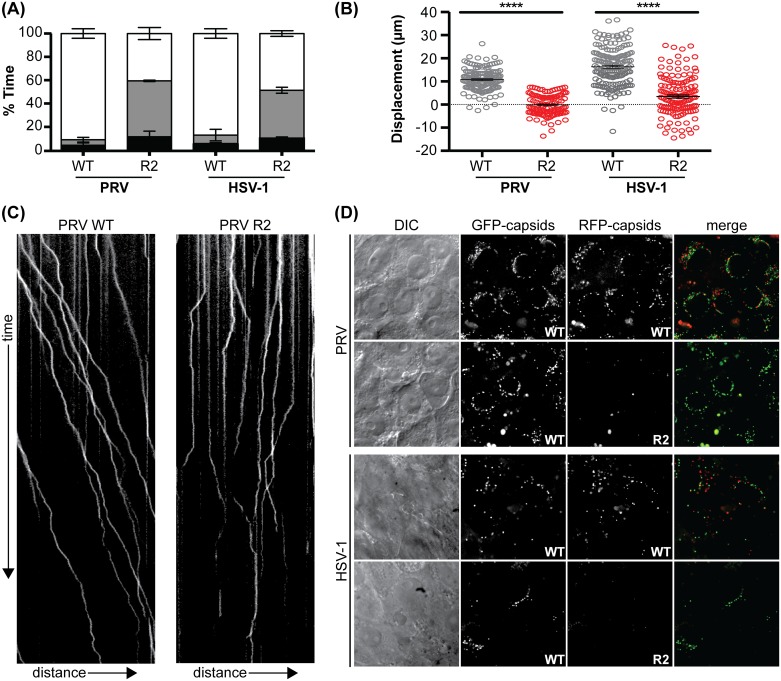
pUL37 R2 is essential for sustained retrograde motion during ingress. Dorsal root ganglion (DRG) sensory neuron explants were infected in 2 ml of media with 3.5 x 10^7^ PFU/ml of PRV (WT and R2 mutant) or 1.3 x 10^7^ PFU/ml of HSV-1 (WT and R2 mutant). Viruses encoded a pUL25/mCherry or pUL25/GFP fusion to provide imaging of individual capsids in living cells. Capsid axonal transport was recorded by time-lapse fluorescence imaging between 3–4 hpi. More than 90 capsids were analyzed per experiment across three biological replicates. **(A)** Fraction of time that individual wild-type (WT) and R2-mutant (R2) capsids moved retrogradely (white), anterogradely (gray), or were motionless (black). Error bars are s.d. **(B)** Net displacement of capsids over a period of 10 seconds. Positive values indicate movement towards neuronal soma (retrograde displacement). Error bars are s.d. (****, p < 0.0001 based on two-tailed unpaired *t* test). **(C)** Representative kymographs of axonal transport in neurons. Distance and time are represented on the x and y axis respectively. **(D)** Delivery of capsids to nuclear rims at 3–4 hpi following co-infection of DRGs with a pUL25/GFP tagged wild-type (WT) virus and either WT or R2 mutant virus encoding a pUL25/mCherry capsid tag.

Our results suggested that the PRV R2 mutant was impaired for microtubule-based transport, which is absolutely required for neuronal infection via long-distance axonal transport [50]. Therefore, studies in primary cultured neurons were next pursued. To expand the scope of these studies to additional alpha-herpesviruses, equivalent mutations were introduced into HSV-1 (Fig 3A). As was observed with PRV, the resultant HSV-1 UL37 R2 mutant propagated to wild-type titers but had a reduction in plaque diameter (Fig 3B and 3C) [47]. To determine if retrograde transport was impaired in primary sensory neurons, live-cell microscopy was used to examine capsid dynamics in axons during initial infection. Whereas wild-type HSV-1 and PRV capsids preferentially moved in the retrograde direction in axons (towards the neural soma), capsids of the R2 mutants moved in an aberrant bidirectional manner best described as “ping-ponging” (S1 Movie). Ping-pong motion was associated with near-zero net displacement of the capsids (Fig 4A–4C).

To test whether the loss of sustained retrograde transport prevented capsid trafficking to neuronal nuclei from axon terminals, dorsal root ganglia (DRG) were cultured *ex vivo* as intact explants and co-infected with equal amounts of wild-type and R2 mutant viruses encoding eGFP- and mCherry-capsids, respectively. Under these conditions, the sensory neurons were maintained in their surrounding tissue but had extended axons that were exposed to the inoculum, which permitted a comparative assessment of the ability of the two viruses to deliver capsids to nuclear rims following retrograde axonal transport [30]. Only capsids from the wild-type HSV-1 or PRV arrived at nuclear rims of neurons within the explants (Fig 4D). This result reinforced the inability of the R2-mutant viruses to traffic retrogradely in axons.

**Fig 5:**
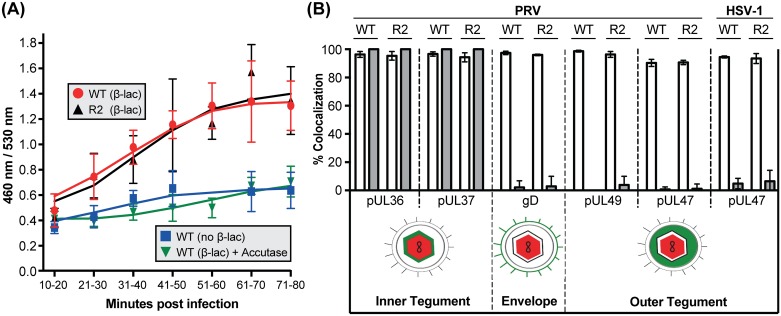
Mutation of R2 does not interfere with virion entry or disassembly in axons. **(A)** DRG sensory neuron explants were loaded with the CCF2 beta-lactamase (β-Lac) substrate and infected with either wild-type (WT) or R2-mutant (R2) PRV encoding β-Lac fused to the pUL35 capsid protein, or wild-type PRV not encoding β-Lac. As an added baseline control, the wild-type (β-Lac) virus was incubated with Accutase for 2 hours to prevent infection by removing the viral glycoproteins. Ratiometric image pairs were captured at 460 nm and 530 nm using 405 nm for excitation. β-Lac cleavage of CCF2, resulting from virion entry into the cells, was monitored as an increase in the 460:530 nm ratio. Data points represent the average fluorescence intensity ratio from > 60 axons (3 independent experiments each consisting of measurements from 20–30 axons per time point). Values are mean ± s.d. **(B)** Fusion-based entry was indirectly monitored by the disassociation of envelope and outer tegument proteins from capsids using dual-fluorescent tagged recombinants of PRV and HSV-1. Capsids were scored for coincident red/green emissions from extracellular virions (open bars) or from particles moving in axons at 3.5 hpi (solid bars). For the latter, only capsids moving > 2.5 μm were scored, and the percentage of particles that colocalized with eGFP was tallied across three independent experiments with > 3 fields imaged per experiment. Values are mean ± s.d.

Although R2 mutation did not impair entry of PRV into transformed epithelial cells (Fig 2A), we could not rule out that the aberrant motion observed with R2 mutants during neuronal infection represented virions that failed to undergo fusion-based entry into axons, and instead were either residing on the axon surface or within endosomes. To specifically monitor fusion-based virus entry into axons, an enzyme-based assay was developed based on an approach that was used to monitor HIV fusion with T lymphocytes [51]. Primary sensory neurons were loaded with the β-lactamase substrate CCF2, which contains a 7-hydroxycoumarin ring linked to fluorescein via a beta-lactam ring. Excitation of the coumarin group produces Förster resonance energy transfer (FRET) to the fluorescein group and emission of green florescence at 518 nm. β-lactamase prevents FRET by cleaving the beta-lactam ring and liberating coumarin fluorescence at 447 nm [52]. To trigger CCF2 cleavage, recombinants of PRV were produced encoding β-lactamase fused to the pUL35 capsid protein (β-lac-capsid). Fusion-based entry of the recombinant viruses was monitored in CCF2-loaded primary sensory neurons. Deposition of the β-lac-capsid into the cytosol exposes the β-lactamase to the cytosolic CCF2 substrate resulting in reduced FRET. As controls, neurons were infected either with PRV that did not encode for beta-lactamase or with beta-lactamase-expressing PRV that was first treated with Accutase (Thermo Fisher) to remove the ectodomains of viral glycoproteins that are essential for entry. The two controls provided a combined baseline for the ratiometric FRET measurements over the experimental time course. The findings supported that wild-type and R2-mutant PRV induced CCF2 cleavage at an equivalent rate, and indicated that mutation of R2 did not impair the kinetics of entry into neuronal cells (Fig 5A).

Herpesvirus entry into cells is coupled with a selective disassembly of tegument proteins from the capsid. To confirm that mutation of R2 did not impair virion disassembly, a collection of dual-florescent viruses that encode mCherry-capsids and eGFP fused to protein constituents of the envelope and tegument was produced. These viruses permitted monitoring of loss of the eGFP marker as an indirect indication of fusion-based entry of individual virus particles in axons [15,53]. Similar to wild-type viruses, R2 mutant capsids moving in axons lacked the gD envelope protein and outer tegument proteins that dissociate from capsids upon entry. Furthermore, mutation of R2 did not compromise the normal retention of pUL36 and pUL37 on capsids following entry (Fig 5B; S2 Movie). Taken together, these findings demonstrated that virus entry and disassembly in neurons was unimpaired by R2 mutation, and further indicated that the capsids displaying aberrant ping-pong motion resided within the axon cytosol.

### The origin of capsids mid-axon in the absence of R2

Paradoxically, R2 mutant capsids displaying ping-pong motion accumulated throughout the length of axons in the *ex vivo* DRG cultures. This was unexpected given that virus entry typically occurs at axon terminals, and the R2 mutant phenotype should prevent the capsids from migrating beyond the point of entry [54]. Therefore, the origin of these capsids was scrutinized using neurons cultured in microfluidic chambers, such that exposure to the virus was restricted to the distal regions of the axons near the terminals. Under these conditions no capsids of the R2 mutant were observed mid-axon, away from the site of inoculation, confirming that R2 mutant capsids were incapable of net retrograde displacement (S3 Movie). We infer that the presence of R2 capsids initially observed throughout the length of axons was due to unrestricted entry of virus particles mid-axon.

### Anterograde axonal transport during egress occurs by a R2-independent mechanism

**Fig 6:**
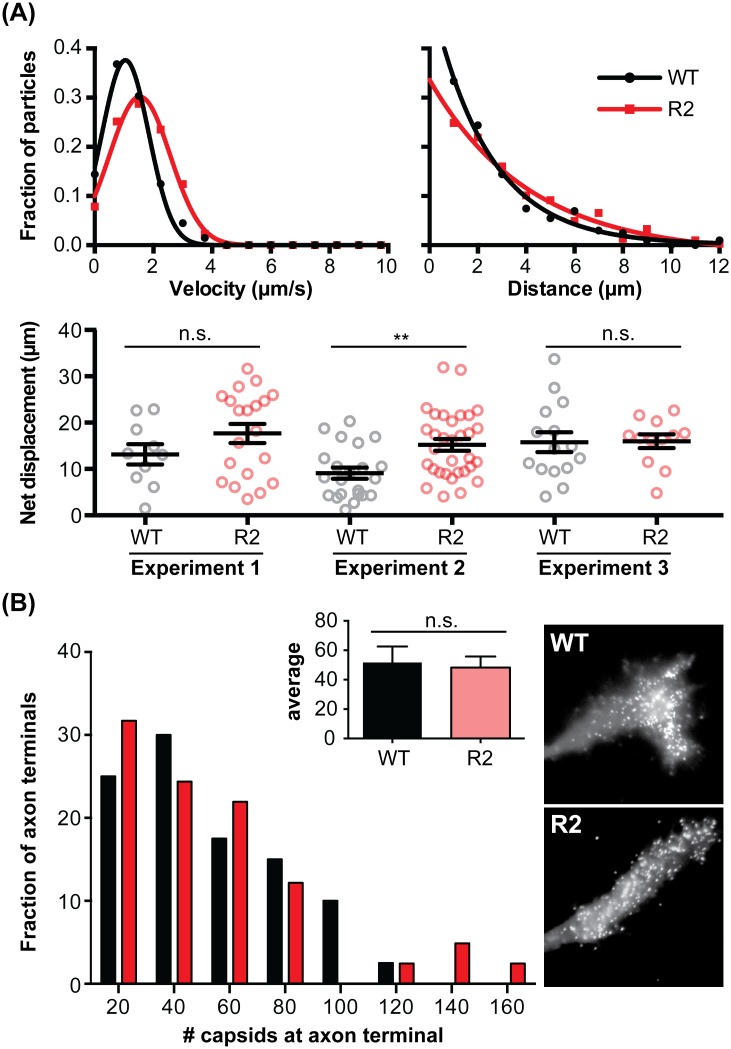
The pUL37 R2 region is dispensable for PRV anterograde axonal transport in culture. Dissociated DRG neurons were infected in 2 ml of media with 2.5 × 10^6^ PFU/ml of either wild-type PRV (WT) or R2-mutant PRV (R2) encoding a pUL25/mCherry capsid fusion. **(A)** Egressing capsids were imaged by time-lapse fluorescent microscopy from 10–13 hpi. Capsids were tracked in isolated axons that were unambiguously contiguous with an identifiable neuronal soma, thereby ensuring that all transport included in the analysis was anterograde directed. More than 30 capsids were tracked per virus per experimental replicate. Three independent experimental replicates were completed and capsid velocities and run lengths for each replicate determined. Gaussian (velocity) or decaying exponential (run length) curves were fit by nonlinear regression (r^2^ > 0.97 for all four curve fits). The net displacement represents the total distance away from the soma that individual capsids moved over a 10 sec recording (bottom panel). Values are mean ± s.d. (**, p < 0.01; n.s., not significant based on two-tailed unpaired *t* test). **(B)** The number of capsids in axon terminals at 17–18 hpi was quantified across three independent experiments with at least 10 terminals examined per virus per experiment. Inset graph indicates average capsid number per axon terminal. Values are mean ± s.e.m. (n.s., not significant based on two-tailed unpaired *t* test). Representative images of capsid accumulation at axon terminals are shown at right.

Alpha-herpesvirus rely on bi-directional microtubule-based axonal transport during initial and late infection, with preferential dynein-based retrograde transport during ingress switching to biased kinesin-based anterograde motion during egress [55]. To determine if R2 was a global regulator of microtubule transport, DRG were dissociated and cultured as isolated neurons, exposing the neuronal soma to the virus inoculum and removing the requirement for retrograde axonal transport to reach the nucleus. Under these conditions, the neurons were productively infected with the PRV R2 mutant and subsequent progeny viral particles were found to transport in the anterograde direction to distal axon terminals equivalently to the wild-type virus (Fig 6). These findings demonstrate that, as in transformed epithelial cells, R2 was not required for the delivery of capsids to nuclei over short distances and was also dispensable for long-distance anterograde transport in axons during egress.

### Alpha-herpesvirus neuroinvasion requires R2

**Fig 7:**
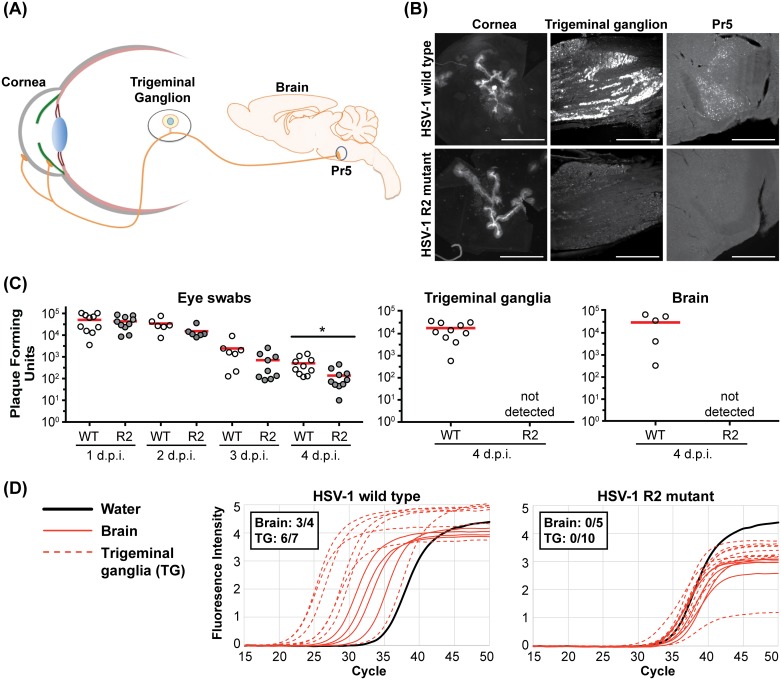
pUL37 R2 is essential for HSV-1 neuroinvasion. **(A)** Diagram of the neuroinvasive route examined in mice following inoculation of a scarified cornea with HSV-1. **(B)** Representative images of the cornea (2 dpi), trigeminal ganglia (6 dpi), and principal sensory trigeminal nuclei (Pr5; 6 dpi), following corneal inoculation with wild-type or R2-mutant HSV-1. Infected cells were visualized by virtue of a pUL25/mCherry fluorescent capsid reporter encoded by the viruses (scale bars for trigeminal ganglia and Pr5 are 500 μm; cornea scale bar is 1000 μm). **(C)** Mice were infected on both eyes with untagged wild-type (WT) or R2-mutant (R2) HSV-1 following corneal scarification. Viral titers in the tear films were independently determined from each eye by swabbing at the indicated day post-infection (dpi). At 4 days post corneal inoculation, the combined titer of the left and right trigeminal ganglia and of the whole brain were determined. The mean titer of each data set is indicated by a red bar (5 mice per virus; *, p < 0.05 based on a two-tailed unpaired *t* test). **(D)** qPCR was preformed using primers directed against the HSV-1 UL35 gene. Trigeminal ganglia and brain samples were collected at 4 days post infection and scored as positive for viral DNA if the threshold cycle (Ct) value was below the average Ct value of the water controls by more than two standard deviations (inset). Amplification curves for both HSV-1 WT and R2-mutant infected samples are shown.

Our results with cultured primary sensory neurons predict that the R2 region may serve as an effector of neuroinvasion *in vivo*. To test this hypothesis, we examined the ability of HSV-1 to infect the mouse nervous system following inoculation of peripheral tissues. For HSV-1, mice were infected at the corneal surface and transmission of infection to the trigeminal ganglia (TG) and subsequently to second order neurons in the brain stem was assessed (Fig 7A). In contrast to wild-type HSV-1, the HSV-1 R2 mutant was not detected in either site despite producing equivalent focal infections on the cornea (Fig 7B). To exclude the possibility that the fluorescently-tagged capsid interfered with HSV-1 R2 neuroinvasion, similar experiments were performed using untagged HSV-1 and viral load was examined in tear film (eye swabs), TG, and brain by previously established methods [56,57]. The near wild-type levels of R2 mutant detected in the tears, coupled with the inability to recover R2 mutant virus from the peripheral (TG) and central (brain) nervous system, added support for an essential role for R2 during neuroinvasion (Fig 7C). Alternatively, the R2 mutant virus may be prone to entering a latent state upon reaching the nervous system, and thereby becoming undetectable by the above approaches. To exclude this possibility, TGs were examined for latent virus by harvesting from mice at 14 dpi and co-culturing the tissue with Vero cells. Cells were monitored continuously for signs of cytopathic effect (CPE) for 10 days post explantation as an indication of the emergence of reactivated virus. Whereas Vero cells co-cultured with 80% of the TGs (8 of 10) from mice infected with wild-type HSV-1 became positive, no reactivation was observed from TGs (0 of 8) harvested from mice infected with the HSV-1 R2 mutant. The presence of HSV-1 DNA in the nervous system was also assessed by qPCR. Consistent with the reactivation assay, viral DNA was detected in tissues from mice infected with wild-type HSV-1, but not in tissues from mice infected with the R2 mutant (Fig 7D).

**Fig 8:**
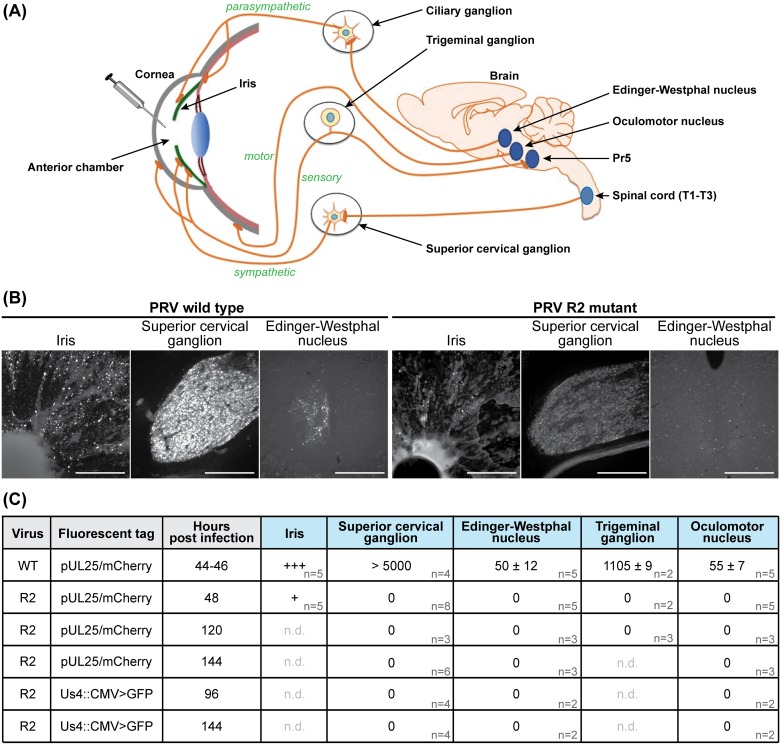
The R2 region of pUL37 is essential for invasion of all retrograde circuitry. **(A)** Diagram of four neuronal circuits exploited by PRV as retrograde transport routes into the rat nervous system. These circuits were each examined following injection of PRV strains encoding a fluorescent capsid reporter into the anterior chamber of the eye. Sensory (trigeminal ganglion), sympathetic (superior cervical ganglion), and parasympathetic (Edinger-Westphal nucleus) circuitry is susceptible to infections following exposure of the cornea, iris, and ciliary body to virus. In addition, motor neurons (oculomotor nucleus) are susceptible to infections resulting from exposure of the extraocular muscles to virus leaked on to the surface of the eye. The parasympathetic circuit includes the ciliary ganglion, which was not examined directly as part of this study but is a mandatory intermediate to observe virus in the Edinger-Westphal nucleus. **(B)** Representative images of indicated tissues at 48 hpi (scale bars = 250 μm). **(C)** Summary of wild-type (WT) and R2-mutant (R2) PRV infection data. Fluorescent cells were counted in each indicated neural tissue (n, number of animals examined; n.d. not determined; values are expressed as mean ± s.e.m.). Cells in the iris were not counted, but infection with the WT (+++) consistently exceeded that of R2 (+).

The link between R2 and neuroinvasion was examined in greater detail in an established rat encephalitic model of PRV infection [42,58]. Firstly, the PRV R2 mutant failed to spread to the TG following inoculation into the anterior chamber of the eye, which was consistent with the HSV-1 R2 mutant defect *in vivo*, and pinpointed R2 as a conserved effector of alpha-herpesvirus neuroinvasion. Furthermore, R2 was essential for retrograde spread of PRV to the parasympathetic ciliary ganglion (CG), sympathetic superior cervical ganglion (SCG), and oculomotor nucleus (OMN) of the midbrain, indicating a loss of neuroinvasive properties in PNS sensory and autonomic neurons, and CNS motor neurons (Fig 8). Combined, these data indicate that R2 is essential for retrograde spread from peripheral tissues to both the PNS and the CNS *in vivo*. To confirm that the failure of the PRV R2 mutant to spread in retrograde circuits was due to a loss of long-distance retrograde axonal transport, as opposed to a block in transmission from mucosal surfaces to innervating axon terminals, PRV was injected directly into the retinorecipient superior colliculus (SC) of the midbrain (S1 Fig). In this paradigm, the injected virus is directly exposed to axons projecting from the retina as well as local neurons. Whereas wild-type PRV had spread retrogradely to the retina by two days post-infection, the PRV R2 mutant failed to spread to the retina (S1 Fig), and remained absent from the retina at five days post-infection.

To confirm that R2 was dispensable for anterograde transport *in vivo*, retinal ganglion cells (RGCs) of rats were directly exposed to the PRV R2 mutant by intravitreal injection. RGCs project axons via the optic nerve to visual centers of the brain, which allows for monitoring of anterograde spread within the CNS (S1 Fig). The PRV R2 mutant spread anterogradely from RGCs to retinorecipient regions of the brain, and furthermore continued to spread via anterograde transneuronal transport to second order nuclei of the visual system (S1 Fig). We conclude that the R2 mutant remained both neurotropic and competent to spread through anterograde circuits within the CNS when the initial requirement for retrograde transport from the periphery was bypassed. Anterograde spread of the R2 mutant through the visual system was delayed but was more comprehensive than that of the wild-type virus, the latter causing a more rapid onset of death that limited its spread. The cause of the slower anterograde spread *in vivo* was not predicted from our studies in cultured neurons, and we suspect that the delay may have manifested from inefficient retrograde transport over short distances in dendrites or the neuronal soma following spread across sites of synaptic contact. This hypothesis is supported by our data in non-neuronal cells, which demonstrated that over short distances R2 promoted efficient gene expression in a microtubule dependent manner (Fig 2B). Taken together, the *in vivo* studies recapitulated the results obtained with primary cultured sensory neurons: R2 is required for retrograde axonal transport but is dispensable for anterograde axonal transport. This phenotype is noteworthy both for its novelty and its utility. For example, an alpha-herpesvirus specifically lacking the ability to transmit through retrograde circuits is a resource for mapping anterograde circuits of the mammalian nervous system.

### Mutation of R2 represents a novel strategy for the development of live-attenuated alpha-herpesvirus vaccines

**Fig 9:**
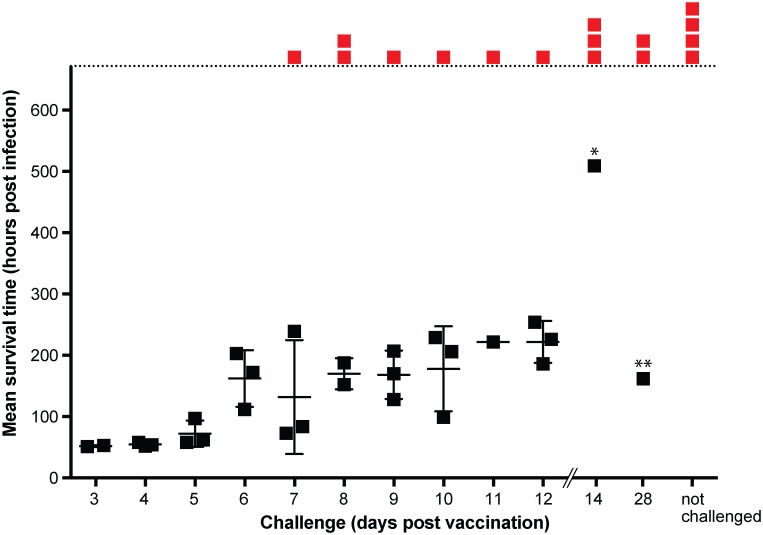
Immunization with the PRV pUL37 R2 mutant protects against lethal encephalitic infection. Mice were vaccinated intranasally with 5 μl of the PRV R2 mutant (6.4 x 10^8^ PFU/ml). Subsequent wild-type challenge was performed by intranasal inoculation with 5 μl of wild-type PRV (8.8 x 10^9^ PFU/ml) at the indicated day post vaccination. Mice showing no signs of disease were euthanized at 672 hours (28 days) post challenge and are indicated in red. * Mouse was euthanized at 504 hours post challenge due to presentation of tremors and an unusual head lesion, although subsequent histology found no indication of viral encephalitis. ** Mouse was euthanized at 168 hours post challenge due to weight loss.

The ability of R2 mutant viruses to replicate and spread in peripheral tissues while being unable to engage in long distance retrograde transport to reach sites of latency offers a new approach to vaccine design [5,6]. As a proof of concept, mice were exposed intranasally with a single dose of the PRV R2 mutant and challenged at varying days post vaccination with 10,000 times the 50% lethal dose of wild-type PRV (S2 Fig). Whereas PRV normally evokes a lethal encephalitis terminating at ~ 2 days, 75% of mice challenged with wild-type PRV at 14 and 28 days post vaccination showed no signs of disease and survived until the end of the experiment at 21 days post challenge (Fig 9). We believe this is the first success at protecting rodents from the unusually high virulence exhibited by PRV that normally causes rapid lethality, and this protection was noteworthy given the extreme circumstances of this high dose challenge and single dose vaccination.

**Fig 10:**
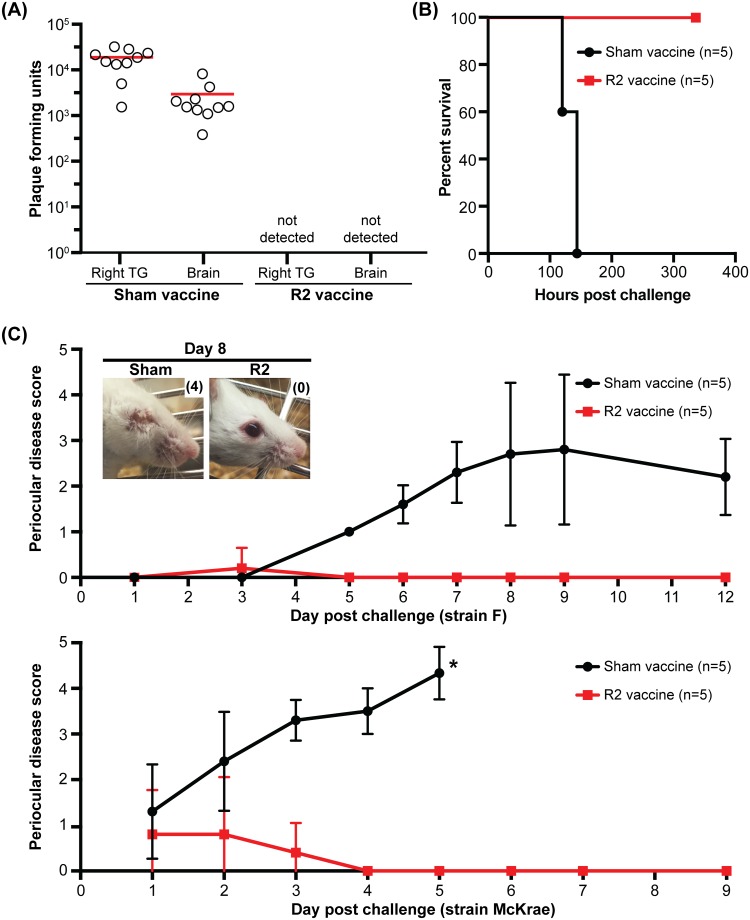
Mice immunized with the HSV-1 R2 mutant are protected from HSV-1 neuroinvasion and periocular skin disease. **(A)** Mice were vaccinated by inoculating the left cornea with 7 μl of the HSV-1 R2 mutant stock (R2; 9 x 10^7^ PFU/ml). Sham vaccinated animals received an equivalent volume of conditioned media. At 14 days post vaccination mice were challenged with 7 μl of wild-type HSV-1 strain F (1.0 x 10^8^ PFU/ml) in the right eye. Mice were euthanized at 4 days post infection and the viral load in the whole ipsilateral trigeminal ganglia and brain were determined. The mean titer of each data set is indicated by a red bar (n.d., not detected). **(B)** Mice were vaccinated as described in (A), at 14 days post vaccination mice were challenged with 5 μl of wild-type HSV-1 McKrae (6.0 x 10^8^ PFU/ml) in the right eye and monitored for survival. **(C)** Mice were vaccinated as described in (A), and at 14 days post vaccination mice were challenged in the right eye with wild-type HSV-1 (either strain F or McKrae as indicated). The right eye of vaccinated animals was scored for periocular skin disease at the indicated day post challenge. Scoring was based on previous published criteria [56]: 0, no lesions; 1, minimal eyelid swelling; 2, moderate eye lid swelling; 3, severe eye lid swelling with no periocular hair loss; 4, eyes swollen shut with minimal ocular discharge and periocular hair loss; 5 eyes swollen shut with severe periocular hair loss and skin lesions. Values are mean disease scores ± s.d. * Mice were euthanized at 5 days post challenge due to pronounced neurological symptoms.

To determine if the efficacy of the R2 mutant as a live-attenuated vaccine extended to HSV-1, mice were infected ocularly with a single dose of the HSV-1 R2 mutant and challenged with wild-type HSV-1. Unlike PRV, the strain of HSV-1 used for these studies (strain F) is not highly virulent in mice [59], but nevertheless invades the PNS and CNS following corneal inoculation via TG sensory neurons (Fig 7A) [60]. Mice vaccinated with the HSV-1 R2 mutant and challenged with wild-type HSV-1 strain F had no detectible virus in either the PNS or the CNS, demonstrating effective protection of the nervous system from HSV-1 invasion (Fig 10A). Furthermore, the protection offered by R2 vaccination prevented lethal disease associated with infections by the more virulent McKrae strain of HSV-1 [61] (Fig 10B). We also note that mice vaccinated with the HSV-1 R2 mutant were protected from the development of periocular disease following wild-type challenge (Fig 10C) [62]. Importantly, the R2 vaccine itself did not cause periocular disease, which is consistent with previous studies that have suggested periocular skin infection requires zosteriform spread from the cornea to periocular skin via sensory ganglia [63]. While further studies are required to assess the immune response that results from R2 vaccination, we hypothesize that the ability of R2 mutant viruses to replicate effectively in peripheral tissues produces an adaptive immune response that effectively blocks the ability of wild-type viruses from invading the nervous system. Achieving this degree of protection reinforces a prior expectation that effective vaccination against HSV would require an attenuated strain that remains fully competent for propagation while having a selective loss of neuroinvasive potential [5].

## Discussion

Few viruses routinely enter the mammalian nervous system, which generally requires crossing the blood-brain barrier or transmission of virions from peripheral tissues to axonal endings and subsequent long-distance retrograde transport in axons [7]. Most neuroinvasive viruses, including rabies virus and poliovirus, reach the neuronal soma by traveling retrogradely in axons within endosomes [64–66]. In contrast, the ability of alpha-herpesviruses to sustain retrograde motion in the axon cytosol suggests that these viruses engage cellular transport motors directly and selectively [30,67]. Upon fusion-based entry into an axon terminal, alpha-herpesvirus transport is bi-directional but is heavily biased for retrograde transport to promote long-distance delivery to the neuronal soma [53,54,68]. The bi-directionality likely results from the ability of capsid/tegument complexes to recruit the dynein/dynactin motor and an opposing kinesin motor [67,69–71]. Our results indicate that the conserved R2 region in the pUL37 inner tegument protein rectifies this motion to produce sustained retrograde bias. *Adenoviridae*, which are rarely neuroinvasive, also bind opposing microtubule motors but unlike herpesviruses do not coordinate motor activity. The stochastic motion produces zero net displacement on average but is sufficient to deliver adenovirus particles to nuclei over short distances [72–74]. The aberrant motion displayed by HSV-1 and PRV R2 mutant viruses is similar to the zero-net displacement natively exhibited by adenovirus, and showcases the added level of control that the alpha-herpesviruses impose on microtubule-based transport: a property that can now be attributed to R2 effector function.

The reliance on R2 for retrograde capsid trafficking in axons, the latter of which can be upwards of a meter in length, is underscored by estimates that in the absence of directed transport it would take a herpesvirus capsid over a century to diffuse 1 cm in the cytoplasm [75]. The requirement for R2 during neuroinvasion was consistent across several paradigms used in this study, which included two related viruses, two rodent infection models, and an avian neuronal culture model. The latter finding deserves some additional commentary due to its utility. Studies of virus delivery from axon endings to the neural soma in culture are often performed in chambers, because the chamber provides a physical barrier that allows for selective virus exposure at the distal axons (S3 Movie). In these systems, extracellular virus cannot diffuse to the neural soma; only virus capable of trafficking retrogradely in axons can reach the soma and productively infect the neuron. The application of the R2 mutant viruses described here establishes that neurons in intact DRG explants are also shielded from extracellular virus inoculum by virtue of the intact tissue architecture, and demonstrate that retrograde delivery examined in explants offers an expedient alternative to traditional chambered systems. R2 mutant viruses can also be considered as controls for traditional cell chamber models, as they can serve to help rule out unintended extracellular diffusion of inoculum due to a leaky chamber.

Because R2 was discovered in part by its conservation, and because we show here that R2 function is conserved across two neuroinvasive herpesviruses of different genera, we expect that all neuroinvasive members of the herpesvirinae are likely dependent on this activity to reach the nervous system of their respective hosts, and therefore to establish life-long latent infections [47]. Although these studies indicate that the mechanism of R2-mediated neuroinvasion is to regulate microtubule transport in axons and guide incoming capsids to neural soma, more work will be needed to elucidate the molecular mechanism of this action. We can infer that R2 is a trafficking factor that coordinates the activities of microtubule motors already bound at the capsid surface, as R2 mutants retain motion that is indicative of microtubule transport by opposing motors. This interpretation is consistent with the finding that the pUL36 tegument protein, which tethers pUL37 to the capsid surface, is the effector that recruits the dynein motor following entry into cells [30]. The requirement for capsids to coordinate opposing motors during initial infection was not necessarily expected, and indicates that either a kinesin motor is effectively performing an antiviral function, which pUL37 overcomes, or a kinesin motor contributes to the guidance of capsids to their target nuclei in the neural ganglia. Deciphering this will require the identification of the kinesin recruitment proteins on the capsid surface.

Whereas several activities were previously mapped to the C-terminal half of pUL37 [44–46,76], R2, along with R1 and R3, are located in the N-terminal half of pUL37. Mutants of all three were attenuated in *vivo*, despite being dispensable for propagation to wild-type titers in cultured epithelial cells (Fig 1) [47]. These findings are suggestive that the N-terminus of pUL37 may be a multifunctional effector of neuronal infection. It is noteworthy that mutation of R2 did not impair the anterograde transport of newly assembled viral particles to distal axon terminals. This result reinforces previous findings indicating that retrograde and anterograde axonal transport occurs by distinct mechanisms [53]. Potential contributions of R1 and R3 include modulating the C-terminal half of pUL37 and its interactions with cellular transport effectors including dystonin/BPAG1, and the intracellular targeting of capsids during ingress and egress phases of neuronal infection [20,77,78]. Understanding the precise contributions of R1 and R3 to infection will require follow up studies of the R1 and R3 mutants in cultured neurons and *in vivo*.

Beyond increasing our understanding of how alpha-herpesviruses invade the nervous system, the production of mutant viruses specifically lacking the capacity to transport retrogradely in axons has several practical applications. First, R2 mutant viruses can be used to specifically trace anterograde circuits within the mammalian nervous system. This new tool, in combination with preexisting mutant viruses that possess retrograde specificity, can now be used to precisely map multi-synaptic circuits within the CNS [79]. The low virulence of R2 mutants has the added benefit of allowing for high-order mapping of neurons. We are currently examining this potential in more detail. Second, R2 mutation may improve the safety of oncolytic herpesvirus vectors by impeding their transport away from the site of injection in brain tumors, thereby restricting lytic activity to the intended malignant tissue. Third, as demonstrated here, R2 mutants have the potential to serve as effective live-attenuated vaccines.

Recent attempts to develop a live-attenuated HSV vaccine have been based on broadly compromising viral propagation [80], or by attempting to abrogate the ability of the virus to invade the nervous system while retaining replication in non-neuronal cells. The latter approach includes mutant viruses that lack expression of specific envelope proteins that play auxiliary roles in HSV entry. Encouragingly, HSV lacking the gE envelope protein fails to spread to mouse dorsal root ganglia following inoculation of abraded skin, and protects from subsequent wild-type challenges [81]. However, gE-null HSV-1 retains baseline neuroinvasive properties and is competent to spread from epithelia to neurons in culture models, albeit at a reduced frequency [82]. In a related approach, HSV-1 encoding a truncated gK envelope protein inefficiently enters cells, including neurons, and when combined with a truncated pUL20 membrane protein renders the virus incapable of causing disease in mice [83]. The decreased capacity of the gE and gK/pUL20 mutant viruses to infect the nervous system is not fully understood and does not appear to be a conserved property of these proteins in related herpesviruses, but nevertheless show exciting potential [84,85].

Our motivation for performing an initial exploration of the pUL37 R2 mutant as a vaccine candidate was based on: (1) its inability to invade the nervous system both in animals and culture despite robust propagation in peripheral tissues and epithelial cell culture, (2) its clearly defined defect that accounts for the ablated neuroinvasive phenotype, and (3) the conservation of the phenotype across the two neuroinvasive herpesvirinae genera. The application of this approach to PRV showcases the unique potential of these recombinant viruses as vaccines. Current live-attenuated PRV vaccines for use in pigs retain sufficient virulence to be lethal in mice [86]. In contrast, the PRV R2 mutant is avirulent in mice, yet offers protection against lethal wild-type PRV challenges. Wild-type PRV is particularly virulent in mice, more so than HSV-1 or HSV-2, and provides a stringent test case of vaccine efficacy. For this reason, R2 mutation may offer a platform for the development of vaccines against many neuroinvasive herpesviruses. The inability of the R2 mutants to infect the peripheral nervous system makes them unable to establish latent infections, while replication in peripheral tissues is benefited from maintaining the integrity of genes required for normal propagation outside of the nervous system. We hypothesize that the robust propagation at the periphery allows for an adaptive immune response of sufficient magnitude to protect mice from a challenge of highly neuroinvasive wild-type HSV-1 or PRV following a single exposure to the R2 mutant strain.

The potential to prevent the establishment of life-long alpha-herpesvirus infections following vaccination may offer a way forward to developing prophylactic vaccines against several noteworthy clinical and veterinary alpha-herpesvirus pathogens. HSV-1 and PRV are representatives of the two principle branches of neuroinvasive herpesviruses, and we expect that this level of protection should extrapolate well to other alpha-herpesviruses. In particular, we anticipate that live-attenuated vaccines based on R2-deficient viruses may offer long sought-after protection against HSV-1 and HSV-2, thereby reducing the incidence of herpes simplex keratitis, herpes simplex encephalitis, and life-threatening neonatal infections [87–89].

## Materials and methods

### Production of the UL37 R2 mutant construct for structural analysis

The plasmid pJP55 encodes the PRV UL37N-R2 mutant gene in frame with a cleavable N-terminal His6-SUMO tag. This plasmid was constructed using 2 rounds of splicing by overlap extension (SOE) PCR using the plasmid pJP23 (WT PRV UL37N) as template in the first round [47], and the intermediate plasmid (pJP56) as the template in the second round. For the first round of SOE-PCR, creating the Q324A, D362A, and R365A mutations, the 5’ region was amplified using the outer forward primer 5’-GCAACCGGTGTTTGGGAA**GCA**GTTGCAGCAAGCGCA-3’ and reverse primer 5’-GCAACCAACTGC**TGC**CTGTGC**TG**CGGTCAGACGAGG-3’, while the 3’ region was amplified using the forward primer 5’-CCTCGTCTGACCG**CA**GCACAG**GCA**GCAGTTGGTTGC-3’ and the outer reverse primer 5’-CGCAAGCTTCTAGGCTGCGCTGGTCGGTGC-3’ (restriction sites are underlined and mutated nucleotides are in bold). Next, the PCR fragments generated above were mixed and used as template for the next step of SOE-PCR using the forward primer of the 5’ reaction and the reverse primer of the 3’ reaction. The PCR product from the last step of SOE PCR was then sub-cloned into pJP23 using the AgeI and HindIII restriction sites generating the intermediate plasmid pJP56. To introduce the H421A and H425A mutations, a second round of SOE PCR was carried out using pJP56 as template. The overlapping primers used for the first step of the second round of SOE-PCR were the reverse primer for the 5’ region 5’-CGGTGCCATACA**TGC**ACGAACAAC**TGC**TTCAACACCTTT-3’ and the forward primer for the 3’ region 5’-AAAGGTGTTCAA**GCA**GTTGTTCGT**GCA**TGTATGGCACCG-3’. The outside primers (forward for 5’ region and reverse for 3’ region) remained the same as in the first round of SOE-PCR. These same outside primers were used to complete the second step of SOE-PCR yielding the UL37N-R2 mutant gene containing all mutations. Similar to the first round of mutagenesis, the resulting PCR fragment from the second step of SOE-PCR was sub-cloned into the pJP23 plasmid using the restriction sites AgeI and HindIII and sequence verified yielding the plasmid pJP55.

### Protein expression and purification

UL37N-R2 protein was expressed in T7 Express *E*. *coli* (New England BioLabs) and purified according to the protocol previously used to produce WT UL37N [47]. Purified UL37N-R2 was concentrated to 3.8–4.0 mg/ml using an Ultra-15 30-kDa cutoff concentrator (Millipore) and stored at 4°C in 20 mM PIPES [pH 7.0], 50 mM NaCl, and 0.1 mM TCEP.

### Crystallization, data collection, and structure determination

Crystals of UL37N-R2 were grown by vapor diffusion at room temperature in hanging drops containing 2 μl protein at ~ 4 mg/ml and 2 μl crystallization solution (30–32% PEG1000, 0.3 M Ca(CH_3_C00)_2_ and 0.1 M imidazole [pH 8.0]). Unlike WT UL37N, the UL37N-R2 mutant required a higher concentration of PEG1000 for crystallization. Large plate-like crystals appeared between 2 and 7 days and were harvested 1–4 weeks later. During harvesting, crystals were soaked in well solution containing 10% glycerol for 1–2 minutes prior to flash freezing in liquid nitrogen. Diffraction data were collected at 100 K on beamline 24-ID-C at the Advanced Photon Source at Argonne National Labs and processed in HKL2000 [90]. Whereas the WT UL37N (PDB ID 4K70) crystallized in space group P21 with 2 molecules per asymmetric unit [47], crystals of the UL37N-R2 mutant took space group P2221 with 1 molecule per asymmetric unit (S3 Table). The non-crystallographic 2-fold symmetry axis in the WT UL37N crystals became the crystallographic 2-fold symmetry axis in the UL37N-R2 crystals. Phases were obtained by molecular replacement using a single copy of WT UL37N structure (PDB ID 4K70) [47] as a search template, as implemented in Phaser [91]. The molecular replacement solution was used as a starting model for refinement in *phenix*.*refine* [92] using data to 2.48 Å resolution. Prior to refinement, 9% of the data were set aside for cross-validation, and residues Q324, D362, R365, H421, and H425 were substituted with alanines. The model was refined in *phenix*.*refine* [92] with iterative rounds of rebuilding in Coot [93]. A composite omit simulated annealing map was used to check the model. Refinement included rigid-body refinement, gradient minimization refinement of XYZ coordinates, individual atomic displacement parameter refinement, and refinement of TLS parameters, all as implemented in phenix.refine. The final R_work_ is 17.81% and R_free_ is 23.66%. All relevant crystallographic statistics are listed in atomic coordinates (S3 Table), and structure factors for UL37N-R2 were deposited to the RCSB Protein Data Bank under accession number 5J2Z.

### Mutagenesis of herpesvirus infectious clones

All recombinant PRV (strain Becker) were derived from the pBecker3 infectious clone and are listed in S2 Table [94]. The PRV R1, R2, and R3 mutant viruses were previously described [47]. Variants of the pBecker3 infectious clones encoding a translational fusion of eGFP to envelope or tegument proteins were produced by an additional round of En Passant mutagenesis using the pEP-EGFP-in template (a kind gift from Nikolaus Osterrieder) with previously described primers [16,95]. New EP primers were designed for translational fusion of eGFP to PRV pUL37: 5’- CGTGGATCGGCTCCTGGCCGAGGTCGACGCCGTGTCCAAAGTGAGCAAGGGCGAGGAG and 5’- GGCTGAAATAACACACGCGCGTGGGGGAAAAATCTTTTTACTTGTACAGCTCGTCCATGC. For translational fusion of beta-lactamase to PRV pUL35, a new En Passant template plasmid was first constructed: pEP-Bla-in. This was accomplished by first producing a pUC-based plasmid encoding resistance to kanamycin and chloramphenicol (pGS3670). The kanamycin cassette was then removed by AvrII digestion and self-ligation, resulting in a pUC plasmid only encoding resistance to chloramphenicol (pGS3672). This latter plasmid served as the vector for cloning a partial duplication of the beta-lactamase coding sequence containing an *aphA*I gene (kanamycin resistance) and an I-SceI cleavage site between the duplicated sequences. This was achieved by digesting pBR322 with HindIII and PstI to remove a portion of the beta-lactamase coding sequence, and ligating in a PCR product of an oversized copy of the removed fragment. The PCR was performed with primers 5’ CC-AAAGCTTATCGATGATAAGCTGTC (reproduction of the vector HindIII site is underlined) and 5’ GCGATGCAT-AGATCTTTATCAGCAATAAACCAGCCAG (NsiI and BglII sites underlined). The NsiI site derived from the second primer was used to clone the PCR product into the vector PstI site, producing the desired duplication within the beta-lactamase coding sequence along with a BglII restriction site at its center (pGS3552). The duplicated beta-lactamase with I-SceI + kanamycin insertion was then PCR amplified in its entirety using primers 5’ GGAAGCTTGCTCACCCAGAAACGCTG (HindIII site underlined) and 5’ CGGGATCCCCAATGCTTAATCAGTGAGGC (BamHI site underlined), which was then cloned into pGS3672 cut with HindIII + BamHI, resulting in pGS3681. Finally, the aphAI gene and I-SceI cleavage site cassette from pEP-EGFP-in was subcloned into the unique BglII site by PCR amplification using primers carrying 5’ BglII sites, resulting in pEP-Bla-in. En Passant mutagenesis was performed with primers 5’- GGGCGCGCACAGACGCGCGCTCCCCGCCGAGCCATCATGGCTCACCCAGAAACGCTG and 5’- CTGCGCGGTGATCGTCCGGGGATTGTTCGGGTCGAAGGACCAATGCTTAATCAGTGAGGC off of pEP-Bla-in to insert beta-lactamase into PRV UL35.

The HSV-1 R2 mutant was derived from a variant of the HSV-1 strain F infectious clone, pGS5923 [96,97]. For the latter, codon changes were introduced through three rounds of En Passant mutagenesis. The first set of primers, 5’- CTGCGAGGCGGTCGGCCTGTCGGGGGGCGTTCTGAGCGCGACGCTGGCGGCTATCATGGGTCCGGCCAGGATGACGACGATAAGTAGGG and 5’- CCGCAGGCTCGCCAGATGTTCCGTCGGCACGGCCGGACCCATGATAGCCGCCAGCGTCGCGCTCAGAACGCCCCAACCAATTAACCAATTCTGATTAG, were used to introduce the Q511A/R515A mutations and produced pGS6151. The second set of primers 5’-GCGGCGGGCGTCCCCGCGCGGACGCCCGCCGGCCACGGACTCGGCGCAGTCCAGGCGCTCTTTGGGTGCATTAGGATGACGACGATAAGTAGGG and 5’-CAGACCAAACACGTTCGACCCCGCGAGGGCAATGCACCCAAAGAGCGCCTGGACTGCGCCGAGTCCGTGGCCCAACCAATTAACCAATTCTGATTAG, were used to introduce the E452A/Q55A mutations into pGS6151 to produce pGS6207. The third set of primers 5’- CAGCAGCGGCTGCTGGCTCTGCTGCAGCAGACGTGGACGTTGATCGCGAATACCAATTCGCCCAGGATGACGACGATAAGTAGGG and 5’- AGCGTCGATCAGGGTGTTGATCACCACGGAGGGCGAATTGGTATTCGCGATCAACGTCCACGTCAACCAATTAACCAATTCTGATTAG, were used to introduce the Q403A mutation into pGS6207 to produce the completed R2 mutant: pGS6264. A variant of pGS6264 encoding a translational fusion of the mCherry tag with the pUL25 capsid protein was produced by an additional round of En Passant mutagenesis as previously described, resulting in pGS6298 [98]. Variants of pGS4553 and pGS6298 infectious clones encoding a translational fusion of eGFP to pUL47 were produced by En Passant mutagenesis using primers 5’-GGTAGCGGACATCCGATAACCCGCGTCTATCGCCACCATGGTGAGCAAGGGCGAG and 5’-TGGATGCGCGCCTCCTGCGCCCCGCGGGTTCGCGAGCCGACTTGTACAGCTCGTC.

### Cell lines and virus propagation

Vero (African green monkey kidney epithelial, ATCC), and PK15 (pig kidney epithelial, ATCC) cells were grown in DMEM (Dulbecco’s Modified Eagle Medium, Invitrogen) supplemented with 10% BGS (bovine growth supplement, HyClone), and were confirmed mycobacterium free and authenticated by the source. BGS levels were reduced to 2% during infection. All PRV strains were produced by electroporation of pBecker3 derivatives into PK15 cells as previously described [13]. The harvested virus was passaged once more on PK15 cells at low multiplicity to create a working stock. Titers of working stocks were obtained by plaque assay on PK15 cells as previously described [26]. HSV-1 strains were produced by electroporation of infectious clones into Vero cells. Using an ECM 630 electroporation system (BTX Instrument Division, Harvard Apparatus) cells were pulsed once with the following settings: 220V, 950μF, 0Ω. Serum levels were reduced to 2% BGS approximately 12 h after electroporation. Virus was harvested at a time at which 100% of the cells displayed pronounced cytopathic effect (CPE) (typically 3–5 days post electroporation).

For single-step growth curves, HSV-1 was harvested from Vero cells and supernatants at 2, 5, 8, 12, and 24 hours post infection [99]. Titers were determined by plaque assay on Vero cells overlaid with 2 ml methocel media (DMEM supplemented with 2% BGS and 10 mg/ml methyl cellulose) and allowed to expand for four days. Plaque diameters were measured using Vero cells plated in 6-well trays infected with approximately 100 plaque forming units (PFU) per well. Images of at least 30 isolated plaques from each infection were acquired with a Nikon Eclipse TE2000-U inverted microscope fitted with a 0.30 numerical aperture (NA) 10 × objective and RFP filter set. To determine the plaque diameter, the average of two orthogonal diameter measurements was calculated for each plaque using ImageJ software [100]. Plaque diameters were expressed as a percentage of the diameter of wild-type HSV-1, which was always measured in parallel. Data sets were plotted using GraphPad Prism 6 (GraphPad Software Inc.).

### Primary neuronal culture and microfluidic chambers

Dorsal root ganglia (DRG) from embryonic chicken (E8-E9) were cultured for 2 to 3 days on poly-DL-ornithine- and laminin-treated coverslips in 2 ml of F12 media (Invitrogen) containing nutrient mix: 0.08 g/ml bovine serum albumin fraction V powder (VWR), 0.4 mg/ml crystalline bovine pancreas insulin (Sigma-Aldrich), 0.4 μg/ml sodium selenite (VWR), 4 μg/ml transferrin (Intercell Technology) and 5 ng/ml nerve growth factor (NGF; Sigma-Aldrich). In a subset of experiments, DRG were cultured in polydimethylsiloxane (PMDS) chambers prepared using an epoxy mold (kindly provided by Eran Perlson, Tel Aviv University) [64]. Chambers were placed on plasma-cleaned coverslips treated overnight with poly-DL-ornithine and subsequently overnight with laminin. Laminin solution was replaced with F12 media prior to the plating of a single DRG explant in the proximal well adjacent to the chamber microgrooves (see illustration within S2 Movie). The explant was cultured in the chamber for 3 days to allow axons to grow to the distal channel. Infection was performed by replacing the F12 media in the distal wells and channel with 7.0 x 10^7^ PFU PRV. Time-lapse imaging of mCherry and eGFP emissions was achieved by automated sequential capture using 100 ms exposures for each channel between 0.5–1.0 hpi.

### Virus penetration kinetics

PK15 cells were grown to confluence in 12-well plates. At the time of the assay, cells were placed on ice and washed once with PBS prior to infection with PFU/well of PRV diluted in serum free media. Cells remained on ice for 1 hour and were then moved to 37°C until processing at the indicated times post infection. During processing the viral inoculum was removed and replaced with either 2 ml of PBS or 2 ml of a citrate solution (40 mM citrate, 10 mM KCl, 0.135 M NaCl [pH 3]). Cells were incubated at room temperature for 1 minute at which time the PBS was removed and replaced with 2 ml methocel media. For citrate-treated wells the citrate was removed and cells washed twice with PBS prior to addition of methocel media. Cells were then incubated at 37°C for an additional 3 days and the number of plaques that formed were counted. For each time point the percent internalization was calculated using the equation: (No. of plaques on citrate-treated well/No. of plaques on PBS-treated well) × 100. Three independent experimental replicates were performed and the results were plotted using GraphPad Prism 6.

To measure penetration kinetics into primary sensory neurons, an adaptation of a beta-lactamase assay was developed. Chick dorsal root ganglia explants were cultured for 2–3 days as described above. Media was removed and replaced with CCF2/AM Live-Blazer dye solution (Invitrogen): 0.6 μl CCF2/AM, 5.4 μl Solution B, 79 μl Solution C, 15 μl 0.1 M Probenecid, and 500 μl F12 media supplemented with nutrient mix (see above) and NGF. Explants were loaded with the CCF2/AM dye solution for 45 minutes at 37°C then washed three times with F12 media. The CCF2-loaded explants were infected with 8.0 x 10^7^ pfu/ml of the indicated recombinant PRV strains. Axons were imaged from 10–75 minutes post infection using an inverted wide-field Nikon Eclipse TE2000-U microscope fitted with a 60x/1.4 NA objective, a Photometrics CoolSnap HQ2 camera, and a Beta-lactamase Ratiometric filter set (Chroma). Sequential images were captured using DIC, followed by excitation with a HQ405/20x filter and capture with HQ460/40m and HQ530/30m emission filters. 100 ms exposures were used for all images. To avoid unintended bias, CCF2 cleavage was quantitated by drawing a region of interest (ROI) along an axon on the DIC image, which was then transferred to the 460 nm and 530 nm images. The average fluorescence intensity of the 460 nm ROI was divided by the corresponding average fluorescence intensity of the 530 nm ROI. Ratiometric values from 20–30 ROIs were recorded for each time point. This procedure was repeated for three independent experiments. The MetaMorph software package (Molecular Devices) was used for image analysis.

### Virus gene expression

PK15 cells were grown to confluence in 6-well plates and infected with PRV at a MOI of 1. Total RNA was isolated at 4 hpi with TRIzol (Invitrogen) according to the manufacturer’s instructions. Contaminating DNA was removed with RQ1 RNase-free DNase (Promega). RNA concentration was determined by absorbance at 260 nm with a NanoDrop 8000 (Thermo scientific). Gene-specific complementary DNA (cDNA) was synthesized using the SuperScript III First-Strand Synthesis System (Invitrogen). SYBR Green-based quantitative real-time PCR was performed on cDNA in a LightCycler 480 II (Roche). All reactions were carried out in 10 μl volumes: 2 μl cDNA, 5 μl LightCycler 480 SYBR Green I Master (Roche), 0.5 μl forward primer and 0.5 μl reverse primer (0.5 μM each), and 2 μl water. The running conditions and primer sequences were based on a previous study [101]. A control sample lacking the reverse transcriptase enzyme was included in parallel. Specificity of primer binding was confirmed by melting point analysis. The crossing points (CP) for each transcript was determined by “Fit Point Method” using LightCycler 480 II software 1.5.0 SP3 (Roche). IE180 mRNA levels were normalized to S28 rRNA, and the fold change of between R2 and WT PRV infections was averaged across three independent experiments. A subset of cells was treated with 9 μM nocodazole for 1 hr prior to infection and maintained in nocodazole throughout the 4 hr infection.

### Fluorescence microscopy and image analysis

Virus transport dynamics were monitored in primary DRG explants. Explants were infected in 2 ml of media with 3.5 x 10^7^ PFU/ml of PRV (WT and R2 mutant) or 1.3 x 10^7^ PFU/ml of HSV-1 (WT and R2 mutant) from 3–4 hpi. Time-lapse images were captured using an inverted wide-field Nikon Eclipse TE2000-U microscope fitted with a 60x/1.4 NA objective and a CascadeII:512 electron-multiplying charge-coupled device (EM-CCD; Photometrics). The microscope was housed in a 37° environmental box (In Vivo Scientific). Moving particles were detected by time-lapse fluorescence microscopy in the red-fluorescence channel at 10 frames per second (continuous 100 ms exposures) for 100 or 500 frames. Particle trajectories were traced in the 100 frame time-lapse image stacks using a multi-line tool with a width of 20 pixels and average background subtraction, and a kymograph was produced using the MetaMorph software package (Molecular Devices). The multi-line tool was again employed to trace kymograph paths, and the fraction of time that a particle was stopped, moving anterograde, or moving retrograde was calculated for each particle. Graphs were created in GraphPad Prism 6.

Virus composition in axons of DRG explants was monitored using dual-fluorescent viruses encoding pUL25/mCherry and either eGFP-pUL47, pUL49-eGFP, gD-eGFP, pUL36-eGFP, or pUL37-eGFP (S2 Table). Time-lapse imaging of mCherry and eGFP emissions was achieved by automated sequential capture with 100 ms exposures for each channel at 3–4 hpi. Red-fluorescent capsids that moved > 2.5 μm were scored for the presence of a coincident eGFP signal. Extracellular viral particle composition was analyzed by collecting supernatants from infected PK15 (PRV) or Vero (HSV-1) cells as previously described [95]. Briefly, cells in 10 cm dishes were infected at a MOI of 5 PFU/cell and incubated for 18 hrs in F12 media lacking phenol red (Gibco) and supplemented with 2% (vol/vol) BGS (HyClone). Supernatants were harvested and cleared of cell debris with a 10 min 3,000 × g centrifugation. Next, 8 ml of cleared supernatant was transferred to a SW41 centrifuge tube and underlaid with a 1 ml cushion of 10% (wt/vol) Nycodenz (Accurate Chemical) in PBS. Samples were centrifuged at 38,500 × g for 60 min. Media and the Nycodenz cushion were gently aspirated from the virus pellet, which was resuspended in 0.1 ml PBS. Prior to imaging, the resuspended virus particles were diluted 1:50 in PBS and 0.07 ml was transferred into a microscope slide chamber consisting of a plasma-cleaned No. 1.5, 22 × 22-mm coverslip. Images were captured using 1.7 s exposures with a CoolSnap HQ2 camera (Photometrics).

Imaging of capsid delivery to nuclear rims of DRG sensory neurons was conducted following co-infections with pUL25/mCherry and pUL25/eGFP encoding viruses. DRG explants were infected in 2 ml of media with 3.5 × 10^7^ PFU/ml of each virus (PRV co-infections) or at 1.0 × 10^7^ PFU/ml of each virus (HSV-1 co-infections). Infected DRG were imaged between 3–4 hpi using a Nikon Ti inverted microscope fitted with a 100 × 1.45 NA objective (Nikon Instruments) and coupled to a CSU-W1 confocal head (Yokogawa Electric Corporation) and a CascadeII:1024 EM-CCD (Photometrics). Illumination was provided by a Sapphire 561 laser (Coherent) and custom laser launch (Solamere Technology Group, Inc.). Nuclei were identified using differential interference contrast imaging.

Anterograde axonal transport following virus replication in DRG sensory neurons was monitored as previously described [55]. Briefly, triturated neurons were seeded on poly-DL-ornithine treated coverslips at low density and cultured for 2 days prior to infection in 2 ml of media with 2.5 × 10^6^ PFU/ml of either wild-type or the R2-mutant PRV. Actively transporting PRV particles were imaged between 10–13 hpi at 10 frames/s (100 ms exposures) for 100 frames, and transport dynamics were measured by kymograph analysis as described above. Particle accumulation at axon terminals was imaged using 1.7 sec exposures on an inverted wide-field Nikon Eclipse TE2000-E fluorescence microscope fitted with a 60 × 1.4 NA objective and a Photometrics CoolSnap HQ2 camera. Particle counts at axon terminals were determined manually across three independent experiments with at least 10 terminals examined per sample.

### Ethics statement

All procedures conformed to NIH guidelines for work with laboratory animals and were approved by the Institutional Animal Care and Use Committee of the University of Nebraska, Lincoln (Protocol: 1086) and Northwestern University (Protocol: IS00003334). Fertilized chicken eggs were obtained from Sunnyside, Inc. and tissue were harvested between embryonic day 8 and 10.

### *In vivo* methods

Intranasal inoculations were performed as previously described [98]. Briefly, male CD-1 mice (6 wk old; Charles River) were maintained for at least 2 weeks under a 12:12 hr light/dark cycle, with 2–3 mice per cage and food and water freely available. Intranasal application of PRV was administered to animals anesthetized by 2.5% isoflurane inhalation. Viral stocks were stored frozen at −80°C and were used immediately after being thawed. Animals received 5 μl of PRV (1.0–6.4 x 10^8^ PFU/ml) in either one or both nostrils. For vaccination studies, animals received 5 μl of the PRV R2 mutant (6.4 x 10^8^ PFU/ml) in each nostril and a second intranasal instillation of 5 μl of WT virus (8.8 x 10^9^ PFU/ml) at 3 to 28 days post vaccination. Behavior was continuously video monitored with images captured every 10 min. Survival times post-inoculation were rounded to the nearest hour. Surviving animals were euthanized at the end of the experiment.

Intraocular injections were performed as previously described [41]. Briefly, male Long Evans rats (8 wk old; Charles River) were maintained two per cage under a 12:12 hr light:dark cycle for at least 2 weeks with food and water freely available. Under isoflurane inhalation anesthesia (2.5%), animals received either an anterior chamber injection of 2 μl of PRV (0.2–4.0 x 10^9^ PFU/ml) or an intravitreal chamber injection of 10 μl of PRV. Injections were performed over a 2 min interval using a Hamilton syringe fitted with a 30 g needle. A fresh stock of virus was thawed for each experiment. Animals were maintained in a biosafety level 2 facility for up to 144 hrs post-inoculation.

Intracranial injections were performed as previously described [40]. Briefly, under isoflurane inhalation anesthesia (2.5%), male Long Evans rats (6 wk old, Charles River) were positioned in a Kopf stereotaxic apparatus (David Kopf Instruments). A craniotomy was performed, and a micropipette attached to a Nanoject II nanoinjector (Drummond Scientific Co.) was lowered to the appropriate position and approximately 0.7 μl of PRV was injected. The micropipette was kept in place for 2 min, elevated 100 μm and another 0.7 μl injected, and kept in place an additional min before being withdrawn. The craniotomy was subsequently packed with gel foam, the incision was sutured, and the animals were monitored in a recovery area until fully mobile. Animals were maintained in a biosafety level 2 facility for 48–102 h post-injection and were prepared for histological analysis. Briefly, under deep anesthesia, animals were intracardially perfused with 0.9% saline followed by 4.0% paraformaldehyde in 0.1 M phosphate buffer (pH 7.3). Retinas were removed and imaged as flat mounts whereas brains were post-fixed overnight in the same fixative containing 20% sucrose and sectioned at 40 μm with a CM1850 cryostat (Leica). Sections were mounted on slides, coverslipped with Vectashield (Vector Laboratories) and imaged with a DM5500B fluorescence microscope (Leica) equipped with a C11440 Orca-flash 4.0 digital camera (Hamamatsu).

Imaging of fluorescently-tagged HSV-1 infected tissues was performed following corneal inoculation of DBA2 mice (6 wk old; Charles River). The animals were anesthetized (2.5% isoflurane inhalation), the corneas gently abraded with a 25-gauge needle, and 5 μl of HSV-1 (6–10 x 10^7^ PFU/ml) was administered to the corneal surface. Two to six days later animals were anesthetized and prepared for histological analyses as described above. Corneas were prepared as flat mounts and imaged. Brains and trigeminal ganglia were post-fixed, sectioned at 40 μm and imaged as described above. In separate experiments, HSV-1 infections of BALB/c mice (9 wk old; Jackson Lab) were carried out in animals anesthetized with an intraperitoneal injection of a ketamine (86.98 mg/kg) and xylazine (13.04 mg/kg) mixture. Each cornea was lightly abraded 10 times in a crosshatched pattern with a 25-gauge needle, and 7 μl of HSV-1 (9 x 10^7^ PFU/ml) was administered to the cornea surface. Prior to infection, the virus stock was sonicated and centrifuged for 2 min at 300 x g to remove cell debris. Eye swabs were collected by lightly anesthetizing the mice with isoflurane, gently proptosing each eye, and wiping a DMEM-moistened sterile cotton swab three times around the eye in a circular motion and twice across the center of the cornea in an “X” pattern. The swabs were placed in 1 ml of DMEM and stored at −80°C. Before titering, the swabs were thawed and vigorously vortexed for 30 seconds. At the indicated day post infection each trigeminal ganglia and the brain were removed and individually homogenized in 1 ml DMEM, sonicated, and stored at −80°C. Titers of recovered HSV-1 from eye swabs and tissues were determined on Vero cells as described above. For reactivation studies whole trigeminal ganglia were harvested and bisected prior to plating on a monolayer of Vero cells. Cells were monitored for CPE for 7 days. If no CPE was observed, cells and tissue were collected, homogenized, sonicated, and 100 μl of the homogenate was plated on a monolayer of Vero cells. Cells were monitored for 10 days for signs of CPE. Tissue was scored positive if CPE was detected from the intact or homogenized tissues.

For HSV-1 vaccination studies, BALB/c mice were inoculated in the left eye with either 7 μl of the HSV-1 R2 mutant stock (9 x 10^7^ PFU/ml), or 7 μl of conditioned media following corneal scarification. Conditioned media was harvested from Vero cells grown in DMEM supplemented with 10% BGS: cells and media were collected together and frozen, and prior to use were thawed, sonicated, and centrifuged to remove cell debris to mimic the handling of the HSV-1 stocks. At 14 days post vaccination mice were challenged with 7 μl of wild-type HSV-1 strain F (1.0 x 10^8^ PFU/ml) or 5 μl of HSV-1 McKrae (6.0 x 10^8^ PFU/ml) by inoculation of the right eye following corneal scarification.

### qPCR on mouse tissues

DNA was isolated from homogenized brain and trigeminal ganglia using the DNeasy Blood and Tissue Kit (Qiagen). For brain samples the DNA was used at a final concentration of 100 ng/μl. For TG samples the final DNA concentration varied between 10–20 ng/μl. Each sample was run in triplicate using a 10 μl reaction volume consisting of: 5 μl of CyberGreen Mastermix (Roche), 0.5 μl forward primer (30 μM), 0.5 μl reverse primer (30 μM), 1.5 μl water, and 2.5 μl of DNA. Run settings were 95°C for 10 min, 50 cycles of 95°C for 15 sec, and 60°C for 30 sec. The forward and reverse primer sequences were previously published [102]: HSV-1 UL35Fwd: GTCTTGGCCACCAATAACTC; HSV-1 UL35 Rev: GGGTAAACGTGTTGTTTGCG; mGAPDHFwd: GATGGGTGTGAACCACGAG, and mGAPDHRev: GTGATGGCATGGACTGTGG.

### Statistical analysis

The statistical tests for all data are justified and the data meets the assumptions of the test. There is an estimate of variation within each group of data, however, the variance is similar between the groups that are being statistically compared. Significance between specific data sets is described in the respective figure legends.

## Supporting information

S1 Movie Aberrant bi-directional motion exhibited by the PRV R2 mutant. DRG sensory neurons were infected in 2 ml of media with 3.5 x 10^7^ PFU/ml of either WT or R2 mutant PRV. Both viruses encoded a pUL25/mCherry fluorescent capsid reporter. Infections were imaged by time-lapse fluorescence microscopy from 3–4 hpi.
(MOV)

S2 Movie Monitoring of viral particle composition in sensory axons. Short time-lapse recordings show the absence of gD-GFP signal (left) and presence of pUL37 (right) on individual axonal capsids. The recordings are representative of the raw data sets that were acquired and used to produce the analysis provided in Fig 5B. Movie is best viewed with continual looping.
(MOV)

S3 Movie The PRV R2 mutant fails to transport in axons in a microfluidic culture chamber. Axon terminals of DRG cultured *ex vivo* in a microfluidic chamber were co-infected with wild-type PRV (encoding pUL25/eGFP) and the PRV R2 mutant (encoding pUL25/mCherry). Frames of alternating eGFP (top) and mCherry (bottom) emissions were captured prior to 1 hpi, with alternating 100 ms exposures from a single field of view mid-axon that was distal from the site of inoculation at the axon terminals.
(MOV)

S1 Fig Mutation of R2 confines neuronal spread of PRV to anterograde circuits. **(A)** Diagram of neuronal circuits examined following PRV injection into either the vitreous humor or the superior colliculus (SC) of rats. Neuronal circuits are shown as black lines with presynaptic terminals indicated by triangles. **(B)** Representative images of the lateral geniculate nucleus (LGN), superior colliculus (SC), olivary pretectal nucleus (OPN), parabigeminal nucleus (PBG), and suprachiasmatic nucleus (SCN) following vitreous humor injection. **(C)** Representative images of the retina and SC following SC injection (all panels are shown at equal magnification; scale bar is 500 μm).
(PDF)

S2 Fig Determination of the lethal infectious dose of PRV in CD-1 mice. Kaplan–Meier presentation of mouse survival following intranasal instillation of wild-type PRV (WT). Viral stock was serially diluted to determine the minimum lethal infectious dose (n = 4 animals for each dose).
(PDF)

S1 Table Amino acid changes encoded by PRV mutated in pUL37 regions 1 through 3 (R1, R2, R3). (PDF)

S2 Table PRV and HSV-1 strains used in this study. (PDF)

S3 Table Data collection and refinement statistics for N-terminus of R2-mutant PRV pUL37. (PDF)
