# Supplementary figures and images for: The pUL37 tegument protein guides alpha-herpesvirus retrograde axonal transport to promote neuroinvasion
Source: PLoS Pathog. 2017 Dec 7;13(12):e1006741. doi: 10.1371/journal.ppat.1006741 (PMC5749899; doi:10.1371/journal.ppat.1006741)

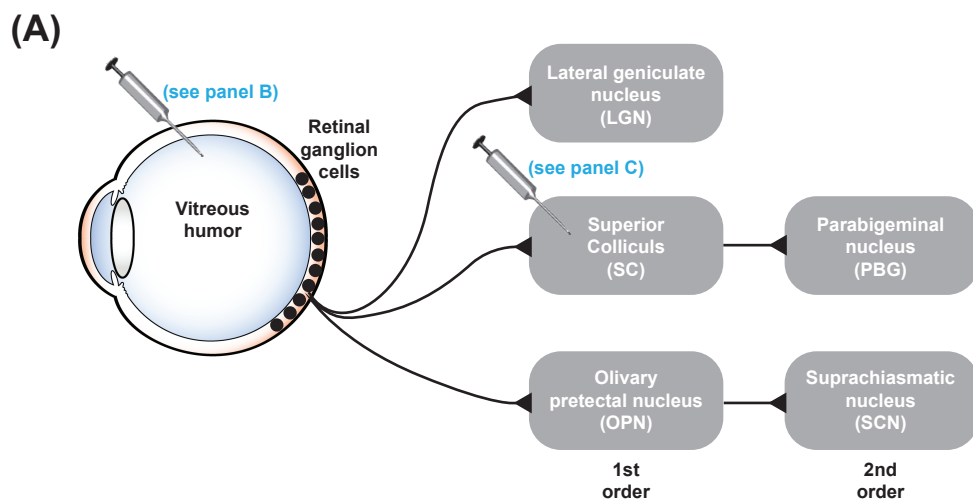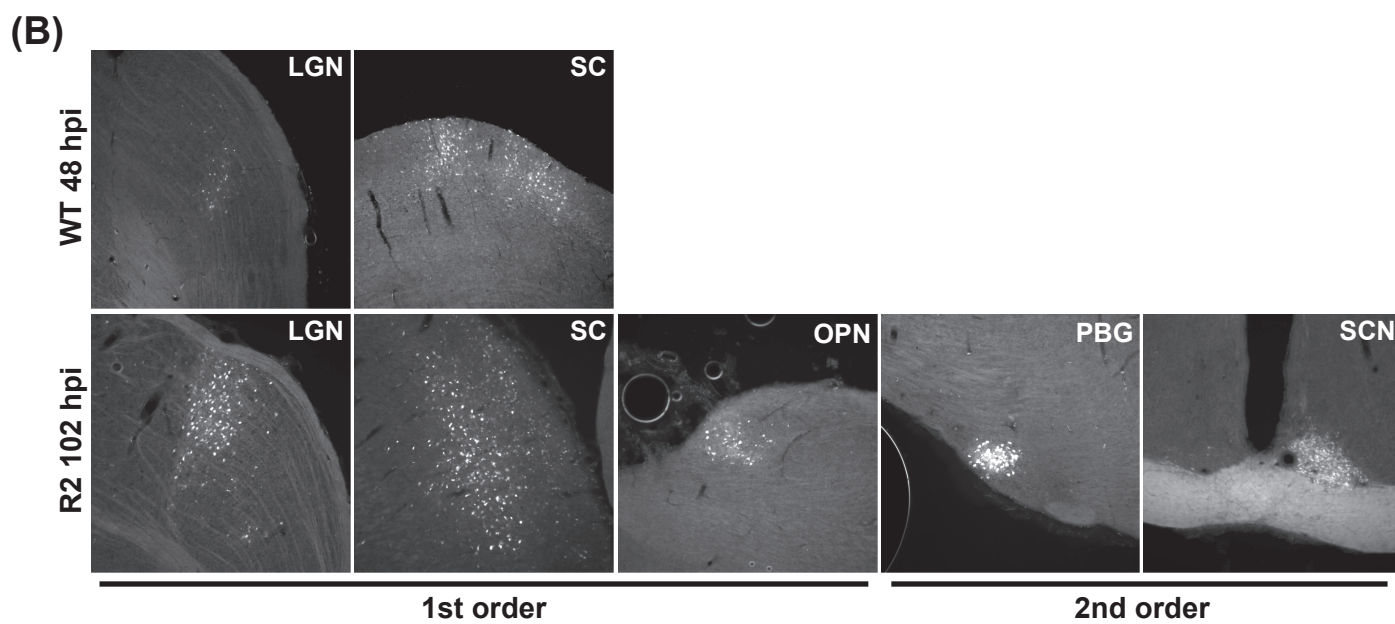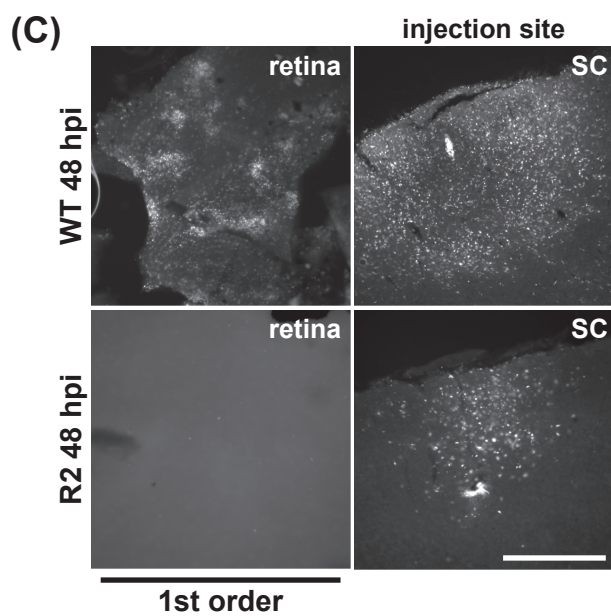

Supplement: S1 Fig — (A) Diagram of neuronal circuits examined following PRV injection into either the vitreous humor or the superior colliculus (SC) of rats. Neuronal circuits are shown as black lines with presynaptic terminals indicated by triangles. (B) Representative images of the lateral geniculate nucleus (LGN), superior colliculus (SC), olivary pretectal nucleus (OPN), parabigeminal nucleus (PBG), and suprachiasmatic nucleus (SCN) following vitreous humor injection. (C) Representative images of the retina and SC following SC injection (all panels are shown at equal magnification; scale bar is 500 μm). (PDF) [file ppat.1006741.s004.pdf]

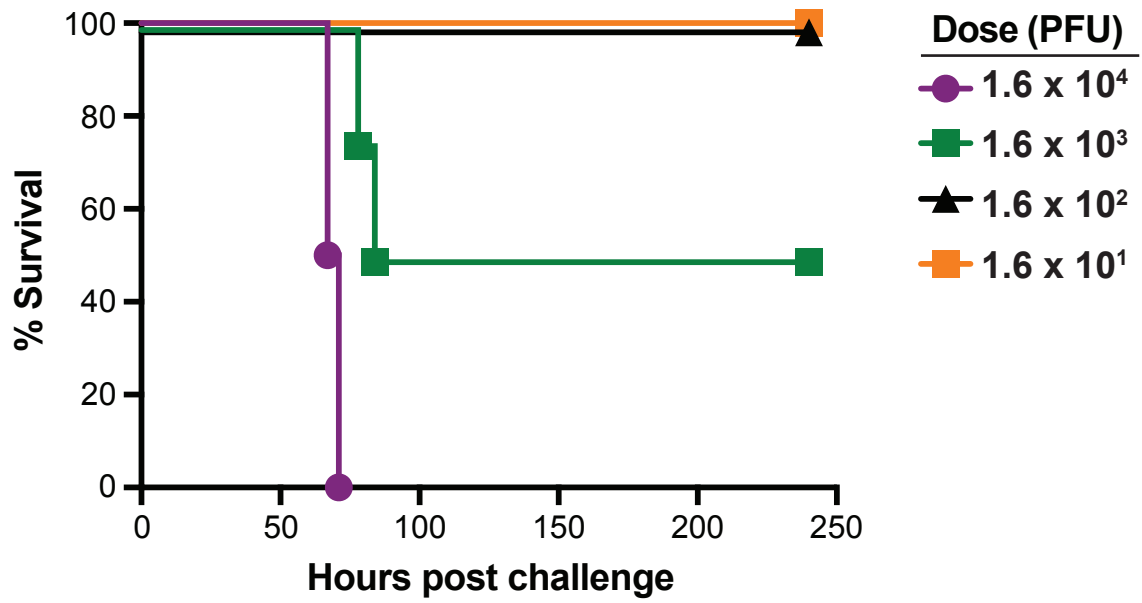

Supplement: S2 Fig — Kaplan–Meier presentation of mouse survival following intranasal instillation of wild-type PRV (WT). Viral stock was serially diluted to determine the minimum lethal infectious dose (n = 4 animals for each dose). (PDF) [file ppat.1006741.s005.pdf]
