# Supplementary material for: The pUL37 tegument protein guides alpha-herpesvirus retrograde axonal transport to promote neuroinvasion
Source: PLoS Pathog. 2017 Dec 7;13(12):e1006741. doi: 10.1371/journal.ppat.1006741 (PMC5749899; doi:10.1371/journal.ppat.1006741)
Supplement: S1 Table — (PDF) [file ppat.1006741.s006.pdf]

Supplemental Table 1: Acid changes encoded by PRV mutated in pUL37 regions 1 through 3 (R1, R2, R3).

pUL37 Allele	Amino Acid Substitutions
R1	V249R / R254A / R285A / D287A / H311A
R2	H421A / H425A / Q324A / D362A / R365A
R3	K203A / P204Q / D239A / E240A / D295A
