# Supplementary material for: The pUL37 tegument protein guides alpha-herpesvirus retrograde axonal transport to promote neuroinvasion
Source: PLoS Pathog. 2017 Dec 7;13(12):e1006741. doi: 10.1371/journal.ppat.1006741 (PMC5749899; doi:10.1371/journal.ppat.1006741)
Supplement: S2 Table — (PDF) [file ppat.1006741.s007.pdf]

Supplemental Table 2: PRV and HSV-1 strains used in this study.

Strain	UL37 allele	Capsid fusion	Tegument fusion	Source
**replication kinetics / axonal transport / neuroinvasion**				
PRV-GS4284	WT	pUL25/mCherry	-	*Bohannon, 2012*
PRV-GS5321	R1	pUL25/mCherry	-	*Pitts, 2014*
PRV-GS5604	R2	pUL25/mCherry	-	*Pitts, 2014*
PRV-GS5350	R3	pUL25/mCherry	-	*Pitts, 2014*
PRV-GS4379	WT	pUL25/eGFP	-	*Bohannon, 2012*
PRV-GS2564	WT	gG::CMV>eGFP	-	This study
PRV-GS6145	R2	gG::CMV>eGFP	-	This study
HSV1-GS5923	WT	-	-	*Richards, 2016*
HSV1-GS6264	R2	-	-	This study
HSV1-GS4553	WT	pUL25/mCherry	-	*Huffmaster, 2015*
HSV1-GS6298	R2	pUL25/mCherry	-	This study
HSV1-GS4677	WT	pUL25/eGFP	-	This study
**virion fusion-based entry and disassembly**				
PRV-GS5179	WT	pUL25/mCherry	gD-eGFP	This study
PRV-GS6032	R2	pUL25/mCherry	gD-eGFP	This study
PRV-GS5507	WT	pUL25/mCherry	eGFP-pUL36	*Huffmaster, 2015*
PRV-GS6105	R2	pUL25/mCherry	eGFP-pUL36	This study
PRV-GS5976	WT	pUL25/mCherry	pUL37-eGFP	This study
PRV-GS6131	R2	pUL25/mCherry	pUL37-eGFP	This study
PRV-GS6063	WT	pUL25/mCherry	pUL47-eGFP	This study
PRV-GS6065	R2	pUL25/mCherry	pUL47-eGFP	This study
PRV-GS6064	WT	pUL25/mCherry	pUL49-eGFP	This study
PRV-GS6066	R2	pUL25/mCherry	pUL49-eGFP	This study
PRV-GS1968	WT	-	-	*Leelawong, 2011*
PRV-GS3800	WT	β-lac-pUL35	-	This study
PRV-GS6366	R2	β-lac-pUL35	-	This study
HSV1-GS6394	WT	pUL25/mCherry	eGFP-pUL47	This study
HSV1-GS6395	R2	pUL25/mCherry	eGFP-pUL47	This study
