# Supplementary material for: The pUL37 tegument protein guides alpha-herpesvirus retrograde axonal transport to promote neuroinvasion
Source: PLoS Pathog. 2017 Dec 7;13(12):e1006741. doi: 10.1371/journal.ppat.1006741 (PMC5749899; doi:10.1371/journal.ppat.1006741)
Supplement: S3 Table — (PDF) [file ppat.1006741.s008.pdf]

Supplemental Table 3: Data collection and refinement statistics for N-terminus of R2-mutant PRV pUL37

	PRV UL37N-R2
Data collection[a]	
Space group	$P222_1$
Cell dimensions	
a, b, c (Å)	51.28, 68.70, 156.23
α, β, γ (°)	90, 90, 90
Resolution (Å)	48.72-2.48 (2.54-2.48)
R_{sym} or R_{merge}	0.126 (0.262)
$I/\sigma I$	14.8 (3.5)
Completeness (%)	96.5 (67.4)
Redundancy	5.6 (2.2)
Refinement	
Resolution (Å)	48.72-2.50
No. reflections (free)	19218 (1719)
R_{work} / R_{free}[b]	0.1781/0.2366
No. atoms	3742
Protein	3610
Solvent	132
B-factors	45.81
Protein	45.94
Solvent	42.25
RMS[c] deviations	
Bond lengths (Å)	0.007
Bond angles (°)	0.779
Ramachandran plot	
Favored (%)	97.69
Allowed (%)	2.31
Outliers (%)	0.0

[a]Values in parentheses are for highest-resolution shell.

[b]R_{work} and R_{free} are defined as $\Sigma ||F_{obs}|-|F_{calc}||/\Sigma |F_{obs}|$ for the reflections in the working or the test set, respectively.

[c]RMS, root mean square.

[d]As determined using Molprobity (molprobity.biochem.duke.edu) [Davis IW, Leaver-Fay A, Chen VB, Block JN, Kapral GJ, Wang X, et al. MolProbity: all-atom contacts and structure validation for proteins and nucleic acids. Nucleic Acids Res 2007 Jul;35(Web Server issue):W375-83. PMID: 17452350]
